# Probiotic Microorganisms in Inflammatory Bowel Diseases: Live Biotherapeutics as Food

**DOI:** 10.3390/foods13244097

**Published:** 2024-12-18

**Authors:** Emanuelle Natalee Santos, Karina Teixeira Magalhães-Guedes, Fernando Elias de Melo Borges, Danton Diego Ferreira, Daniele Ferreira da Silva, Pietro Carlos Gonçalves Conceição, Ana Katerine de Carvalho Lima, Lucas Guimarães Cardoso, Marcelo Andrés Umsza-Guez, Cíntia Lacerda Ramos

**Affiliations:** 1 Post-Graduate Program in Food Science, Federal University of Vale of Jequitinhonha and Mucuri (UFVJM), Street MGT 367—Km 583, No. 5000, Alto da Jacuba, Diamantina 39100-000, MG, Brazil; 2 Post-Graduate Program in Chemistry Engineering, Polytechnic School, Federal University of Bahia (UFBA) and Salvador University (UNIFACS), Street Professor Aristídes Novis, 02, Federação, Salvador 40210-630, BA, Brazil; 3 Post-Graduate Program in Food Science, Federal University of Bahia (UFBA), Barão of Geremoabo Street, s/n, Ondina, Salvador 40171-970, BA, Brazil; 4 Post-Graduate Program in Systems Engineering and Automation, Department of Automatic, Federal University of Lavras (UFLA), University Campus, Lavras 37000-200, MG, Brazil

**Keywords:** Crohn’s disease, ulcerative colitis, smart probiotic, boxplot

## Abstract

(1) Background: Inflammatory bowel diseases (IBDs) are characterized by chronic and complex inflammatory processes of the digestive tract that evolve with frequent relapses and manifest at any age; they predominantly affect young individuals. Diet plays a direct role in maintaining the gut mucosal integrity and immune function. Regarding the diet, the administration of probiotics stands out. The use of probiotics for IBD treatment has shown promising effects on consumers’ quality of life. (2) Methods: This study aimed to conduct a literature review on the effects of probiotic and smart probiotic ingestion on IBD and analyze the available literature based on the searched keywords using boxplot diagrams to search for scientific data in the online literature published up to October 2024. (3) Results: Google Scholar (containing ~6 × 10^6^ articles) and Science Direct (containing ~5 × 10^6^ articles) were the databases with the highest number of articles for the keywords used in the study. When analyzing the content of the articles, although probiotic microorganisms are currently not part of the standard treatment protocol for IBD, these live biotherapeutics have proven to be an effective treatment option, considering the adverse effects of conventional therapies. Furthermore, the development of genetically engineered probiotics or smart probiotics is a promising treatment for IBD. (4) Conclusions: Probiotics and smart probiotics could represent the future of nutritional medicine in IBD care, allowing patients to be treated in a more natural, safe, effective, and nutritious way. However, although many studies have demonstrated the potential of this biotherapy, clinical trials standardizing dosage and strains are still necessary.

## 1. Introduction

The gut epithelium as a semi-permeable barrier that selectively regulates the circulation of molecules. However, some factors like stress, Westernized diets, or the use of antibiotics can alter the composition of the microbiota and thereby impair gut homeostasis [1,2]. High-fat diets modify the gut microbiota composition, which can induce inflammation that may contribute to the development of obesity and inflammatory bowel disease (IBD) [1,2]. IBD is characterized by a chronic and complex inflammatory process affecting the digestive tract, with no known etiology, which evolves with frequent relapses and manifests at any age, though it predominantly affects young individuals. IBD has two clinical forms: ulcerative colitis (UC) and Crohn’s disease (CD) [1,2]. IBDs can assume severe clinical forms and more commonly affect young individuals, leading to a significant impairment of quality of life, high treatment costs, and, frequently, hospitalizations [3]. In CD, the lesion can occur anywhere along the digestive tract, from the mouth to the anus. These lesions are transmural (affecting the entire thickness of the intestinal wall) and discontinuous, with affected areas interspersed with healthy regions [4]. CD may present with deep ulceration, intestinal stenosis, and, in some patients, the presence of internal and external fistulas. The main symptoms of CD include diarrhea, abdominal pain, weight loss, fatigue, low-grade fever, and bloody stools [5,6].

Conversely, UC affects the rectum, extending continuously in a proximal direction and potentially involving the entire colon [1,4,6]. Symptoms of UC include abdominal pain, diarrhea with or without blood, tenesmus, and evacuation urgency. A prevalent characteristic is the presence of nocturnal bowel movements [7]. Complications of UC may include toxic megacolon, massive hemorrhage, and even colon cancer [8,9]. Accurate diagnosis depends on the combined analysis of the clinical history; physical, endoscopic, radiological, and histological findings; and laboratory tests. This detailed evaluation is essential for distinguishing between CD and UC [8,9].

Historically, UC was perceived as a Western disease based on the high incidence and prevalence reported in industrialized countries (especially northern Europe and North America). However, the incidence and prevalence of IBD have continued to increase in several regions around the world in recent decades; many developing countries, traditionally considered low-incidence areas, are now experiencing a dramatic increase in the number of new cases [10,11].

In developing countries, the adoption of a Western lifestyle has become increasingly prevalent, potentially contributing to the rising incidence of IBD and highlighting the role of environmental factors in its development. Although it is considered a region of low IBD prevalence, the true situation may differ due to inadequate public health records. These diseases are not classified as mandatory notifications, and data from patient records and health service files remain insufficient [2,12]. Although the exact mechanism remains unclear, the etiology of IBD may be related to genetic, immunological, and environmental aspects. A dysregulated immune system is associated with responses generated among the gut microbiota [13,14].

The intestinal microbiota (IM) is a diverse and dynamic community comprising commensal bacteria, fungi, and viruses, with bacteria being the largest constituent, representing approximately 1000 different species. The development of the IM begins shortly after birth and continues until a symbiotic relationship with the host is established [13,14]. In a healthy individual, more than 90% of intestinal bacterial species belong to four primary phyla, namely, Bacteroidetes (23%), Firmicutes (64%), Actinobacteria (8%), and Proteobacteria (3%) [14,15]. However, when pathogenic bacteria are predominant, or when the bacterial diversity and stability are disrupted, a condition known as dysbiosis arises [14,15,16].

Dysbiosis leads to an immunological imbalance that triggers intestinal inflammatory responses [17,18,19,20,21,22]. Cytokine imbalance regulated by activated immune cells is an initiating factor in UC, causing diffuse superficial inflammatory lesions [22]. Imbalances in the gut microbiota and consequent dysbiosis are mainly caused by a diet rich in protein and fat and low in fiber but can also be a consequence of the use of some medications or stress [17,18,19,20,21].

The current treatment for UC aims to achieve steroid-free clinical remission, postpone hospitalization and surgery, promote mucosal healing, improve the quality of life, and prevent disability. Mesalazine, containing the active ingredient 5-aminosalicylic acid (5-ASA), is generally used as a first-line treatment for patients with mild to moderate UC [23], achieving a remission rate of approximately 50% [22]. For patients who do not respond to mesalazine, corticosteroids and immunosuppressive agents (such as azathioprine, 6-mercaptopurine, cyclosporine A, and methotrexate) are employed [15]. Most common clinical therapeutic drugs can cause side effects or discomfort. Alternatively, the intake of probiotic-related products affects the intestinal microbiota and is associated with human health [23].

Probiotics are live bacteria and yeasts described as being capable of preventing and controlling mainly gut diseases among other diseases caused in the human body through intestinal dysbiosis [6,16,24,25,26,27,28,29,30,31,32,33]. Several probiotic bacteria/yeast strains are currently used in real-life clinical research to combat symptoms during inflammatory bowel disease outbreaks. Some probiotic microorganism strains, i.e., bacteria from the genera *Lactobacillus*, *Lacticaseibacillus*, *Lentilactobacillus*, *Leuconostoc*, and *Acetobacter*, as well yeasts from the genera *Saccharomyces*, *Kluyveromyces*, *Lachancea,* and *Kazachstania*, can counteract pathogenic bacteria by producing antimicrobial peptides (bacteriocins and bacteriocin-like molecules) [6,16,24,25,26,27,28,29,30,31,32,33].

The adequate administration of probiotic microorganisms tends to promote numerous benefits for the human body including balancing the intestinal microbiota, enhancing immunity, combating allergies, detoxifying the body, and regulating the body for the better absorption of nutrients and vitamins, among other benefits [6,16,24,25,26,27,28,29,30,31,32,33]. Furthermore, treatment with probiotic microorganisms can increase the concentration of short-chain fatty acids in the gut milieu, which play a protective role in maintaining the gut mucosa integrity [34,35,36]. As such, the regular consumption of probiotics can be considered a nutritious live biotherapeutic [6,16,24,25,26,27,28,29,30,31,32,33,34,35,36].

As therapeutic drugs for IBD treatment may cause side effects or some discomfort for patients, biotherapeutics using probiotics have emerged as an interesting alternative. Therefore, the objective of this study was to conduct a literature review to assess the beneficial effects of ingesting probiotic microorganisms against IBD. The reader will be updated on in vivo studies on regular probiotic consumption, as well as provided with an understanding of genetically modified probiotic microorganisms called “smart probiotics.” In addition, the available literature data (the number of articles related to languages and databases) were analyzed using a boxplot to understand where more articles are available. Boxplots are a powerful graphical representation of data that provide an overview and numerical summary of a dataset, allowing for a graphical visualization of the statistical distribution of a variable [37,38,39]. A boxplot is a standardized way of displaying the distribution of data based on a five-number summary (“minimum,” first quartile (Q1), median, third quartile (Q3), and “maximum”). It can show outliers and if the data are symmetric [37,38,39]. In this review, the boxplot technique showed which databases were used to search for articles and which languages were most published.

## 2. Methods

Online databases were used for the preparation of this manuscript: (Latin American and Caribbean Health Sciences (LILACS), PubMed, Medical Literature Analysis (MEDLINE), Science Direct, Google Scholar/Google Academic, Bioline International and Springer Link), using keywords in English, Spanish, and Portuguese. The keywords used in English were “inflammatory bowel disease”, Crohn’s disease”, “ulcerative rectocolitis”, “gut barrier”, “microbiota”, “probiotic”,“IBD”, “dysbiosis”, and “gut”; in Spanish, they were “enfermedad inflamatoria intestinal”, “enfermidade de Cronh”, “retrocolitis ulcerosa”, “barreira intestinal”, “microbiota”, “probioticos”, “IBD”, “disbiosis”, and “del intestino”; and in Portuguese, they were “doenças Inflamatórias intestinais”, “doença de Crohn”, “retrocolite ulcerativa”, “barreira intestinal”, “microbiota”, “probióticos”, “IBD”, “disbiose”, and “intestino”.

Articles published up to October 2024 were included in this study. For the initial selection of articles, abstracts were read considering the objectives, methodology, results, and publication date. Boxplot analysis was used in this study [39]. The data were grouped as follows: (i) repository: this group has 9 clusters, each consisting of the repository with the respective number of articles; (ii) language: this group has 3 clusters, each comprising the language of publication with the respective number of articles; (iii) keyword: this group has 9 clusters, each formed by the article’s keyword (regardless of the publication language) with its respective number of articles.

Boxplot analyses were quantified using MATLAB 2011 software (1994–2024 MathWorks, OPENCADD Advanced Technology, São Paulo, Brazil) to evaluate the number of articles concerning the three groups (repository, language, and keywords). Subsequently, all articles were selected according to their title and abstract. Review articles and in vitro studies that used only cell culture were excluded. Studies using animal models and clinical trials were included. For final selection, the full texts with the following main aspects were evaluated: “Probiotic Microorganisms in Inflammatory Bowel Diseases”, “Smart Probiotic”, and “Probiotics in health”.

## 3. Results

**Figure 1:**
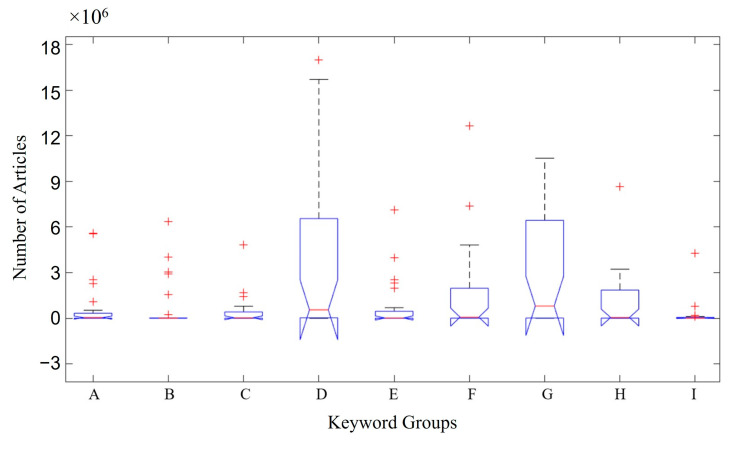
Distribution of the number of articles by keywords. Group A—keyword in English (inflammatory bowel disease); in Spanish (enfermedad inflatoria intestinal); in Portuguese (doenças inflamatórias intestinais). Group B—keyword in English (Crohn’s disease); in Spanish (enfermidade de Crohn); in Portuguese (doença de Crohn). Group C—keyword in English (ulcerative rectocolitis); in Spanish (retrocolitis ulcerosa); in Portuguese (retrocolite ulcerativa). Group D—keyword in English (gut); in Spanish (del colina); in Portuguese (intestino). Group E—keyword in English/Spanish/Portuguese (IBD). Group F—keyword in English/Spanish/Portuguese (microbiota). Group G—keyword in English (probiotic); in Spanish (probioticos); in Portuguese (probiótico). Group H—keyword in English (gut barrier); in Spanish (barrera intestinal); in Portuguese (barreira intestinal). Group I—keyword in English (dysbiosis); in Spanish (disbiosis); in Portuguese (disbiosis). Red cross (**+**) represents atypical values.

Boxplots were used to present the data from this study (number of articles about the keywords, database, and language). Figure 1 shows the ratio between the number of articles and keywords used. Group D contains the words in English (gut), Spanish (del intestinal), and Portuguese (intestino). Group G includes the words in English (probiotic), Spanish (probioticos), and Portuguese (probiótico). This group represents the highest number of articles found (~6 × 10^6^ articles) across the different databases searched. This can be seen in the median number of articles in groups D and G (Figure 1), demonstrating a much larger volume of papers than that in the other groups and followed by Groups F (words in English/Spanish/Portuguese (microbiota)) and H (words in English (gut barrier); in Spanish (barrera intestinal); and in Portuguese (barreira intestinal)), both containing ~2 × 10^6^ articles. Group B (words in English (Crohn’s disease); in Spanish (enfermidade de Crohn); and in Portuguese (doença de Crohn)) was the group with the lowest number of articles at ≤ 0.1 × 10^6^ articles.

**Figure 2:**
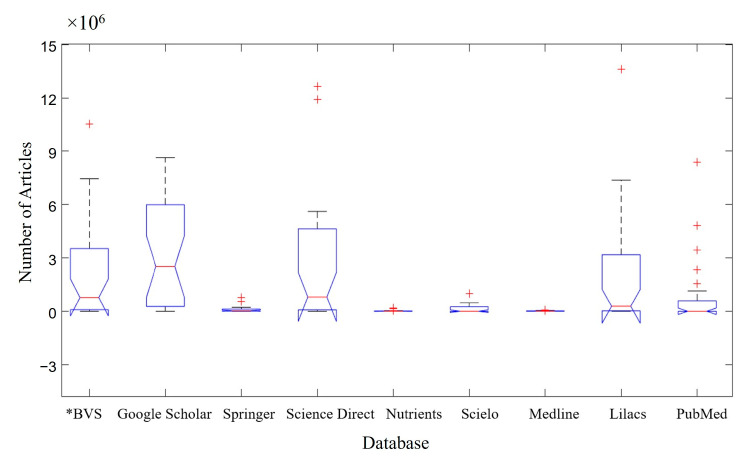
Articles per database ratio. * BVS—Virtual Health Library. Red cross (**+**) represents atypical data.

Figure 2 shows the list of articles by each database. Google Scholar emerged as the database with the highest number of articles available when the keyword searches for all the groups (A–I) in Figure 1 were analyzed, containing ~6 × 10^6^ available articles. This can be seen in the median representation of Google Scholar (Figure 2), which visually demonstrates a much larger volume of papers than those of the other groups. The boxplot technique allows for these analyses, even when the differentiation with the Science Direct database is not as relevant in visual terms. This was followed by the Science Direct database, containing ~5 × 10^6^ available articles, and the BVS—Virtual Health Library and Lilacs databases, both containing ~4 × 10^6^ available articles. By contrast, Nutrients and Medline were the databases with the fewest published articles (≤0.1 × 10^6^ articles). The notch of the “Google Scholar” does not intercept the notch of the other databases searched; therefore, the median of the Google Scholar database is different from the other databases with 95% certainty.

**Figure 3:**
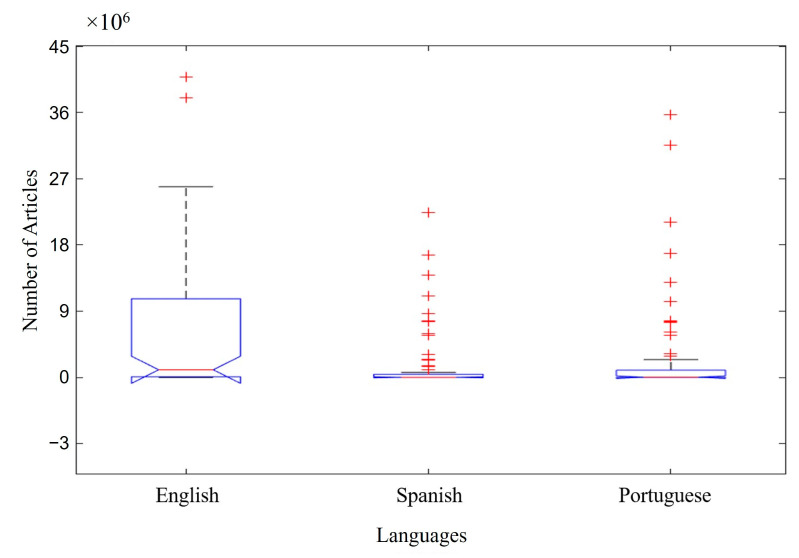
Number of articles by languages searched. Red cross (**+**) represents atypical data.

Figure 3 shows the list of articles for each language searched, considering all the groups of keywords (A–I) in Figure 1 and the databases in Figure 2. The majority of the articles were published in English, totaling ~10 × 10^6^ available articles. In this case, the median was not as convincing in differentiating the data due to the discrepant volume of articles in English compared to Spanish and Portuguese. However, the boxplot technique still allowed for this relevant differentiation when observing the axis of the article number. Portuguese was the second-highest-ranking language for the number of articles, totaling ~0.1 × 10^6^ available articles. The smallest number of articles found was in Spanish, totaling a value of ≤0.05 × 10^6^ available articles.

The two databases with the highest number of articles found were Google Scholar followed by Science Direct. Table 1 and Table 2 show the correlation between the quantitative analysis of the scientific articles published up until October 2024 and the respective databases analyzed. It is possible to observe the prevalence of articles in English (Figure 3).

In the “Google Scholar” database (Table 1), the keyword with the highest number of available articles was “gut” (707 thousand articles), followed by “microbiota” (209 thousand articles) and “inflammatory bowel disease” (143 thousand articles available). In contrast, the Science Direct database (Table 2) showed the largest number of articles for the keyword “gut” (90 thousand articles available), followed by “inflammatory bowel disease” (49.184 thousand articles available) and “microbiota” (46.923 thousand articles available).

Following the boxplot results, 89 articles were selected considering the similarity of the title, abstract, and full text with the main aspects of the manuscript: “Probiotic Microorganisms in Inflammatory Bowel Diseases”, “Smart Probiotic”, “Probiotics in health”. Articles that did not address topics related to the focus of this review were excluded.

## 4. Discussion

Boxplots are powerful graphical tools that provide an overview and numerical summary of a dataset [37,38,39]. In this study, boxplots were used to analyze the number of articles related to languages and databases associated with IBD, microbiota, and probiotics. It was possible to correlate the database to the keywords, language, and the number of articles found. Considering the two databases with the highest number of articles found, Google Scholar and Science Direct, it is possible to observe a correlation between the number of articles published up until October 2024 with all the databases. Articles in English are predominant in the databases studied. The use of the “median” factor was essential for evaluating the graphs and the statistical demonstration of the quantitative differentiation of the data analyzed using the boxplots. Regarding the content of the researched articles, several beneficial associations of probiotic intake were found, such as improvement of dysbiosis [40,41,42], epithelial barrier function [43,44], colon shortening [40,41,42], immunomodulation of inflammatory responses [45,46], reduction in the Disease Activity Index (DAI) score [47], and histological scores [47].

### 4.1. Probiotic Microorganisms for IBD: Live Biotherapeutics as Food

The gut epithelium is a semi-permeable barrier that selectively regulates molecules’ circulation. However, factors like stress, Westernized diets, and the use of antibiotics can alter the composition of the microbiota and, thus, gut homeostasis [1,2,48]. High-fat diets modify the gut microbiota composition, which can induce inflammation that may contribute to the development of obesity and IBD [48,49,50,51]. IBD is characterized by chronic gastrointestinal inflammation, presenting cycles of relapse–remission, and can be classified into CD and UC [50]. Therefore, treatments to restore the intestinal microbiota composition and inflammatory response can be extremely important [51].

Food plays a crucial role in maintaining gut mucosal integrity and immune function. The Western diet, characterized by a higher caloric intake and consumption of sugar-sweetened beverages, is negatively associated with gut microbiome diversity. In contrast, a Mediterranean-style diet with a higher consumption of fruits, vegetables, and phytochemicals has been associated with increased gut microbial diversity [3,5,24,28,40,41,42,44,45,46,47,52,53,54,55,56,57,58,59,60,61,62,63,64,65,66,67,68,69,70,71]. As well as this, the administration of probiotics stands out. Several studies have been carried out to analyze the beneficial effects of the administration of probiotics against IBD and its consequences in experimental models of colitis with animals and to a lesser extent in some clinical trials. According to the analyzed articles, it is possible to observe a positive effect of probiotic use on IBD, as observed in clinical trials and animal model studies [3,5,24,28,40,41,42,44,45,46,47,52,53,54,55,56,57,58,59,60,61,62,63,64,65,66,67,68,69,70,71].

Dore et al. (2019) [5] conducted a retrospective cohort study and analyzed the clinical records of 200 patients with IBD. Of these, 78 were diagnosed with CD and 122 with UC. The most used probiotics among the patients were *Lactobacillus* sp., *Streptococcus* sp., and *Bifidobacterium* sp. According to the authors, for the patients using probiotics for a period ≥75% of the disease duration, the need for systemic steroids, hospitalization, and surgery fell to zero events per person-year in the patients with UC. It decreased by 93% in the patients with CD. Another study, conducted in a hospital in China with 40 patients diagnosed with IBD, analyzed the effects of the combined treatment of pentasa (a drug used to treat IBD) and the probiotics *Bifidobacterium* sp. and *Lactobacillus* sp. on the composition of the microbiota and the prognosis of the disease. The patients in the control group received only pentasa and a combination of probiotics. Pentasa was administered to the observation group. The results showed that the combination of probiotcs and pentasa led to an improvement in dysbiosis, reduced DAI scores, and the numbers of some inflammatory indicators such as Lactoferrin, blood α1-antitrypsin, β2-microglobulin, C-reactive protein, IL-6, and IL-4 [8].

Studies conducted by Bjarnason et al. (2019) [71] evaluated the short-term effects of consuming a probiotic (Symprove) dietary supplement against gut inflammation in patients with IBD compared with placebo. Symprove is a probiotic nutritional supplement that contains four bacteria strains: *Lactobacillus rhamnosus* NCIMB 30174, *Lactobacillus plantarum* NCIMB 30173, *Lactobacillus acidophilus* NCIMB 30176 and NCIMB 30175, and *Enterococcus faecium*. This supplement is a suspension prepared in barley extract, with each 50 mL/dose containing approximately 10 billion live bacteria. The placebo was a liquid identical in appearance, taste, and packaging. In this study, the participants answered a quality of life questionnaire (QQL) and were asked to consume 1 mL/kg every morning for 4 weeks on an empty stomach. The efficacy of the supplement was measured by changes in QQL responses, differences in clinical disease activity scores between active and placebo treatment, and changes in laboratory measures, including Fecal Calprotectin (FCAL). Eighty-one patients with UC and sixty-one patients with CD completed the study. The results showed that the FCAL levels were significantly reduced only in the patients with UC. However, no significant changes were observed in the QQL scores and laboratory data between the patients receiving the probiotic versus those receiving a placebo.

Mousavi et al. (2020) [25] show the effects of the probiotic yogurt culture containing *Bifidobacterium lactis* (*B. lactis*) and *Lactobacillus acidophilus* (*L. acidophilus*) on the blood cells of patients with inflammatory bowel disease (IBD). The study found that the treatment with *B. lactis* increased the expression of anti-inflammatory cytokines, such as IL-10 and TGF-β, while reducing the levels of pro-inflammatory cytokines, including TNF-α and IFN-γ. Based on these findings, the authors suggested that the probiotic yogurt culture containing *B. lactis* could be a promising therapeutic candidate for managing IBD.

Different authors have evaluated the beneficial effects of probiotic microorganisms in IBD using animal models. Table 3 provides a summary of these studies [3,24,28,40,41,42,44,45,46,47,52,53,54,55,56,57,58,59,60,61,62,63,64,65,66,67,68,69,70]. The studies were carried out with a Varied Number (VN) of animals, and the most used animal models were C57BL/6 and BALB/C. The most used chemical for inducing IBD was Dextran Sodium Sulfate (DSS), but Trinitrobenzenesulfonic Acid (TNBS) and oxazolone were also employed, albeit less frequently.

According to Table 3, studies using probiotics in animal models have demonstrated their efficacy in reducing inflammation in IBD. The use of probiotics reduced the DAI and histological scores and improved colon shortening. The DAI is a classification on a scale of 0–4, which evaluates weight loss, intestinal bleeding, and stool consistency [7]. Similarly, histological scores are assessed by scoring the intensity of inflammation, the inflamed area/extent, crypt damage, and the percentage of involvement [72].

The gut microbiota produces short-chain fatty acids (SCFAs), such as butyrate, propionate, lactate, and acetate, through the fermentation of non-digestible carbohydrates. These SCFAs lower the pH of the colon, thereby inhibiting the proliferation of pathogens [73]. The analyzed studies indicate that probiotic bacteria, including *Enterococcus faecium* CRL 183, *Lactobacillus helveticus* 416, and *Bifidobacterium longum* [54] or *Lactococcus lactis* ML2018 [66], stimulated the production of short-chain fatty acids. These metabolites play a crucial role in competing with pathogens for nutrients and adhesion sites in the intestine [54,66]. In addition to probiotic bacteria, a variety of probiotic fungi, such as *Saccharomyces, kazachstania,* and *Kluyveromyces,* play important roles in the fermentation of indigestible carbohydrates and protection against pathogens [3,54,66,73]. Other microbial metabolites are also involved in the beneficial effects of probiotics, such as beta-glucan, polyamines, K vitamin, and some B-complex vitamins, which are essential for blood clotting and energy metabolism in the consumer [54,66,73].

Probiotics present in the gut interact with the host’s immune system, contributing to the regulation of the immune response and the maintenance of immunological homeostasis [9,56,57]. Probiotic microorganisms can interact with Payer’s plaques, present in the gut, which results in the stimulation of B lymphocytes, the production of IgA, and the favoring of the nonspecific phagocytic activity of alveolar macrophages. This interaction triggers systemic effects by secreting mediators that activate the immune system [59,60,61,62,63,64,65,66]. Immunomodulation by probiotics through the induction of anti-inflammatory and regulatory responses may be particularly important for their role in protecting against autoimmune and inflammatory diseases. Strains that induced higher levels of the anti-inflammatory cytokine IL-10 and lower levels of pro-inflammatory cytokines such as IL-1β, IL-6, interferon γ (IFNγ), and TNF-α offered protection in induced colitis [9].

In the area of “Mental Health”, probiotics improve mood and reduce symptoms of stress and depression [26,27,33]. Some microorganisms such as *Lactobacillus acidophilus*, *Bifidobacterium infantis*, *Lacticaseibacillus casei*, *Lentilactobacillus lactis*, *Bifidobacterium longum, Kluyveromyces* spp., *Kazachstania* spp., and *Saccharomyces* spp., contribute to the production of neurotransmitters such as serotonin, norepinephrine, and gamma-aminobutyric acid (GABA). These microorganisms can also influence the gut–brain axis, modulating the expression of neurochemical receptors to produce antidepressant and anxiolytic effects [26,27,33].

Some studies have compared the effects of probiotic administration with conventional drug treatments used for IBD. The probiotics *Bifidobacterium bifidum* 231 [56] and ID-JPL934, composed of *Lactobacillus johnsonii* IDCC9203, *Lactobacillus plantarum* IDCC3501, and *Bifidobacterium animalis* subsp. *lactis* IDCC4301 [57], showed similar efficacies to treatment with dexamethasone and sulfasalazine 500 mg, respectively. These results suggest that the administration of probiotics may play a prominent role in the clinical management of IBD.

Clinical trials have investigated the effects of consuming probiotics such as VSL#3 [57], Bifico [57], and improve [71] in combating IBDs. The results showed that the reduction in IBD-related events was more significant in people who consumed probiotics for a longer duration. The beneficial effects of probiotics were observed in improving dysbiosis and laboratory test results.

It is important to highlight that, due to the diversity of species of microorganisms that inhabit the intestine and the ability of some species to synthesize neuroactive molecules, the digestive tract has a rich and highly potential source of pharmacokinetics, which could influence intestinal/body health [3,16,25,26,32,33]. Studies have demonstrated that the use of probiotic microorganisms and a healthy diet containing cereals, vegetables, and fruits, including some prebiotic ingredients [4], can improve the gut microbiome and, consequently, the body’s homeostasis [3,16,25,26,32,33].

**Figure 4:**
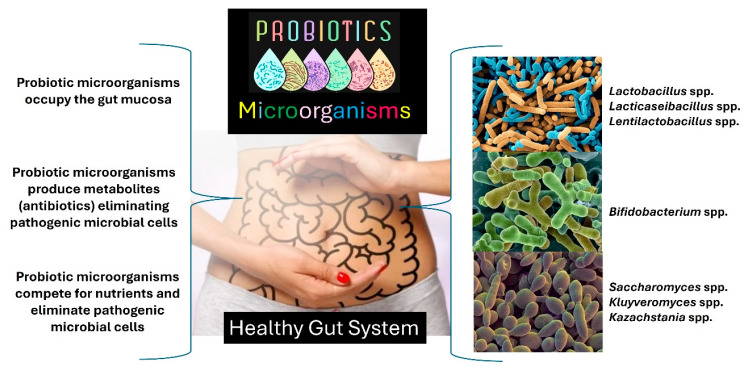
The action of probiotic microorganisms in the gut using animal models. The figure created by the authors is based on scientific literature [3,6,16,25,26,30,31,32,33].

In general, probiotic microorganisms, such as *Lactobacillus* spp., *Bifidobacterium* spp., *Lacticaseibacillus* spp., *Lentilactobacillus* spp., *Bifidobacterium* spp., *Kluyveromyces* spp., *Kazachstania* spp., and *Saccharomyces* spp., act by inhibiting pathogens’ intestinal colonization and combat undesirable and opportunistic microorganisms [3,6,16,25,26,30,31,32,33]. Figure 4 summarizes the action of probiotic microorganisms in the gut as natural biotherapeutics. Ingesting probiotics can help the gut function properly. Probiotic dietary supplements are ideal when they have 1 billion Colony Forming Units (CFUs)/Dose [3,6,16,25,26,30,31,32,33].

### 4.2. Action of Genetically Engineered Probiotic Microorganisms or Smart Probiotics in IBD: Live Biotherapeutics as Food

**Figure 5:**
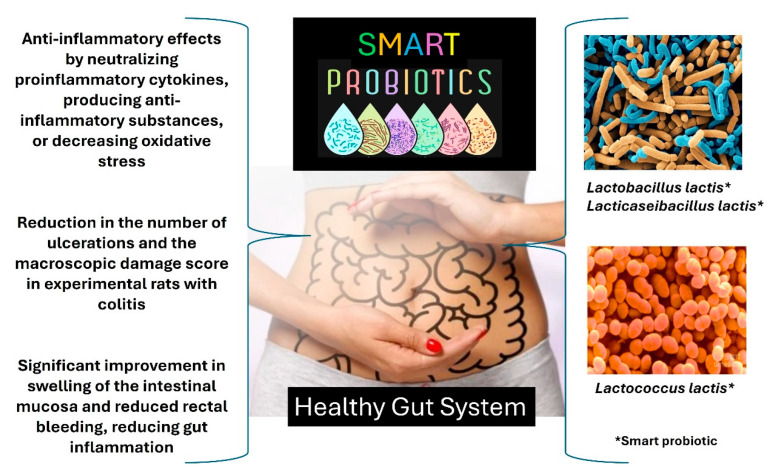
Smart probiotics and their functionalities using animal models. The authors created the figure according to scientific literature [22,34,74,75,76,77,78,79,80].

Genetically engineered probiotic microorganisms, also known as smart probiotics, are live microorganisms. They can respond to external stimuli and provide active metabolites with therapeutic potential in IBD and exhibit anti-inflammatory properties. Therefore, they are also “live biotherapeutics” [34,74]. The presence of recombinant proteins can further enhance the beneficial effects of smart probiotics. Figure 5 shows examples of smart probiotics and their functionalities using animal models as natural biotherapeutics [22,34,74,75,76,77,78,79,80].

**Figure 6:**
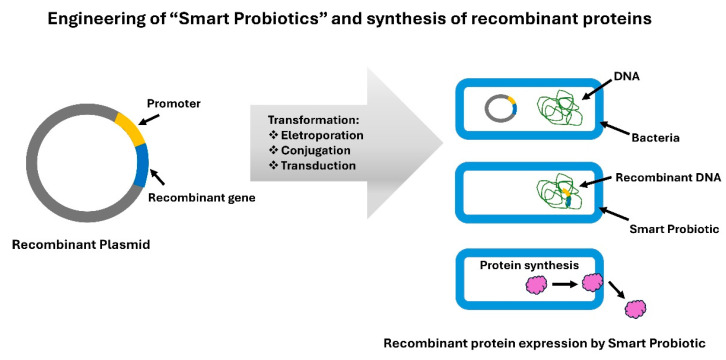
Engineering smart probiotics and synthesis of recombinant proteins. Figure created by the authors according to scientific literature [76,77,78,80,81,82].

Smart probiotics for the treatment and diagnosis of IBD are developed using recombinant DNA technology. This molecular biology technique consists of a heterologous DNA fragment inserted into a plasmid vector (e.g., pGEM-T vector system I (PROMEGA)) [78,80,81]. The recombinant plasmid is then introduced into “super competent” bacteria, such as *Escherichia coli* (JM109), using techniques like electroporation [81]. The heterologous DNA fragment contains genes that express the synthesis of recombinant proteins of interest (Figure 6) [78,80,81].

Alternative methods for producing smart probiotic strains include conjugation and transduction. In conjugation, bacteria share a plasmid containing the heterologous DNA fragment through conjugative pili [76], whereas in transduction, the heterologous DNA fragment sharing is mediated by a bacteriophage [77] (Figure 6).

Molecular biology and genetic engineering enable the development of sophisticated systems to produce genetically engineered probiotic microorganisms or smart probiotics [22,34,74,75,76,77,78,79,80,82]. These advanced probiotic strains show great promise for combating intestinal inflammatory points and delivering therapeutic molecules/metabolites [22,34].

Smart probiotics have been developed to treat various host physiological conditions [22,34,74,75,76,77,78,79,80]. Microorganisms can be genetically modified to act and produce molecules of interest for diagnosing and treating IBD. Scientists manipulate the metabolism of probiotic microorganisms by selectively adding and deleting genes corresponding to enzymatic reactions. When the host ingests the smart probiotics, these microorganisms adhere to the gut. Signals from the host indicating a physiological deficiency bind to the transcription factor of the smart probiotic, forming an activated complex which triggers the expression of the therapeutic-producing gene. The therapeutic molecule produced by the smart probiotic will treat the host symptoms, thereby reducing inflammation and/or active symptoms [22,34,74,75,76,77,78,79,80]. Smart *Lactobacillus lactis*, *Lacticaseibacillus* lactis, and *Lactococcus lactis* were engineered to act on colorectal cancer. These smart bacteria bind to the heparan sulfate proteoglycan on active cancer cells and secrete an enzyme called myrosinase to inhibit the activities of these cancer cells. Smart probiotics have also been used to kill *Enterococcus* spp. infection in the gut by producing antimicrobial metabolites [22,34,74,75,76,77,78,79,80].

Previous studies have shown the role of probiotic microorganisms in IBDs, and evidence reports that the gut microbiota is a key factor in modulating the host immune response [3,22,24,28,34,40,41,42,44,45,46,47,52,53,54,55,56,57,58,59,60,61,62,63,64,65,66,67,68,69,70,71,72,73,74,75,76,77,78,79,80,81,82]. Despite promising results in preclinical models performed with mice, one must consider that the mice gut microbiota differs from the microbiota present in the human gut [3,22,24,28,34,40,41,42,44,45,46,47,52,53,54,55,56,57,58,59,60,61,62,63,64,65,66,67,68,69,70]. The difference between human and mice gut microbiota may lead to genetic mutations or a reduction in the growth rate of smart probiotics due to the different gut environments [3,22,24,28,34,40,41,42,44,45,46,47,52,53,54,55,56,57,58,59,60,61,62,63,64,65,66,67,68,69,70]. Another crucial point concerns the specificity and safety issues of these genetically engineered microorganisms or smart probiotics [76,77,78,80,82]. Studies on smart probiotics are gaining a significant increase, making them a promising and futuristic technology [76,77,78,80,82].

Although significant progress has been made in the development of new probiotic strains [83,84,85,86,87,88,89] and smart probiotics, there is still a need for future in vitro, in vivo, and clinical studies [3,22,24,28,34,40,41,42,44,45,46,47,52,53,54,55,56,57,58,59,60,61,62,63,64,65,66,67,68,69,70,74,75,76,77,78,79,80,82]. Even so, current advances in developing live biotherapeutics indicate that smart probiotics are promising treatments for IBD [22,34,74,75,76,77,78,79,80,82,83]. Smart probiotics could represent the future of nutritional medicine in IBD care, allowing patients to be treated more naturally, safely, effectively, and nutritiously.

## 5. Conclusions and Prospects

The application of boxplots to investigate the available literature on the use of probiotic microorganisms against IBD showed that the keywords “intestine” and “probiotics” had the highest number of articles among the terms searched. In addition, the Google Scholar database, followed by Science Direct, presented the highest number of articles related to IBD, microbiota, and probiotics published up until October 2024. The predominant language of the articles was English.

When analyzing the content of the articles, although probiotic microorganisms are currently not part of the standard treatment protocol for IBD, these live biotherapeutics have proven to be an effective treatment option, considering the adverse effects of conventional therapies. Studies have highlighted the benefits of administering probiotic microorganisms against colitis in animal models. The treatment of IBDs through this therapy has shown promising effects since research on a wide variety of strains, both single and in combination, is increasingly advanced worldwide.

Furthermore, current advances in the development of live biotherapeutics indicate that smart probiotics are promising treatments for IBD. Smart probiotics could represent the future of nutritional medicine in IBD care, allowing patients to be treated naturally, safely, effectively, and nutritiously. Additionally, there is a promising possibility of producing new probiotic/functional foods and beverages. Still, as prospects, the genetic engineering methods used to develop synthetic smart probiotics have great potential for treating and preventing different types of human pathogenesis. Smart probiotics may become a new method and food for treating GI disorders, neurological disorders (e.g., dementia, Parkinson’s, Alzheimer’s), and various forms of cancer.

However, additional clinical studies involving humans are still needed to test the efficacy of known probiotic strains, novel strains, and smart probiotics, since these studies are less frequent than studies in animal models. Therefore, more studies are still needed to establish the doses and formulate a guideline for each probiotic strain to be used in IBD as live biotherapeutics.

## Figures and Tables

**Table 1:** Number of articles published up until October 2024 analyzed in the “Google Scholar” database.

Language	Keywords	Number of Articles
English	Inflammatory bowel disease	143.000
	Crohn’s disease	17.000
	Ulcerative rectocolitis	265
	Gut barrier	47.600
	Microbiota	209.000
	Probiotic	57.600
	IBD *	42.500
	Dysbiosis	32.300
	Gut	707.000
Spanish	Enfermedad inflamatoria intestinal	16.500
	Enfermidade de Crohn	658
	Retocolitis ulcerosa	78
	Barreira intestinal	16.900
	Microbiota	209.000
	Probióticos	13.800
	IBD *	0
	Dysbiosis	2.810
	Del intestino	23.000
Portuguese	Doenças inflamatórias intestinais	16.800
	Doença de Crohn	3.510
	Retocolite ulcerativa	1.750
	Barreira intestinal	13.200
	Microbiota	17.200
	Probióticos	5.500
	IBD *	0
	Disburse	1.470
	Intestino	19.300

* IBD—inflammatory bowel disease.

**Table 2:** Number of articles published up until October 2024 analyzed in the “Science Direct” database.

Language	Keywords	Number of Articles
English	Inflammatory bowel disease	49.184
	Crohn’s disease	15.168
	Ulcerative rectocolitis	39
	Gut barrier	26.570
	Microbiota	46.923
	Probiotic	21.517
	IBD *	20.185
	Dysbiosis	11.111
	Gut	90.675
Spanish	Enfermedad inflamatoria intestinal	1.898
	Enfermedad de Crohn	1.902
	Retocolitis ulcerosa	39
	Barrera intestinal	813
	Microbiota	46.923
	Probioticos	300
	IBD *	0
	Disbiosis	174
	Del intestino	2.078
Portuguese	Doenças Inflamatórias intestinais	181
	Doença de Crohn	285
	Retocolite ulcerativa	1
	Barreira intestinal	124
	Microbiota	24.701
	Probióticos	300
	IBD *	0
	Disbiose	17
	Intestino	2.387

* IBD—inflammatory bowel disease.

**Table 3:** Studies using probiotic microorganisms and their beneficial effects on IBD using animal models.

Reference	Probiotic Microorganisms	Principle Characteristics	Key Benefits
	**Bacteria**		
[52]	*Lactobacillus reuteri* R2LC or *L. reuteri* ATCC PTA	Traditional preparation of fermented food/beverage; diluted powder or capsule consumption	Reduced DAI ^1^, histological score, MPO ^2^, IL-1b, and IL-6. Increased mucus thickness. *L. reuteri* R2LC increased occludin and ZO-1 in crypts.
[46]	GI7 ^3^	Diluted powder or capsule consumption	Reduced weight loss, histological score, inflammation, hemorrhage, TNF-α ^4^, IL-6 and IL-1β. Prevention of colon shortening.
[53]	*Lactobacilluscasei* (Lbs2)	Traditional preparation of fermented food/beverage	Reduced macroscopic characteristics, histological score, IL-12, IL-17, and TNF-α; increased IL-10 and TGF-beta ^5^.
[41]	*Bacillus subtilis*	Diluted powder or capsule consumption	High doses showed increased survival. Reduced necrosis, weight loss, intestinal shortening, dysbiosis, IL-12, IL-17, IL-23, and LPS ^6^ concentration; increased IL-10 and restored tight junctions.
[54]	*Enterococcus faecium*, *Lactobacillus helveticus*, *Bifidobacterium longum*	Traditional preparation of fermented food/beverage; diluted powder or capsule consumption	In the gut, no visible ulcers or swollen areas were observed; reduced severity of colitis and weight loss; improved intestinal microbiota and production of short-chain fatty acids (propionate and acetate).
[55]	*Lactobacillus paracasei* (LS2)	Traditional preparation of fermented food/beverage	Reduced weight loss, MPO activity, and levels of cytokines IL-6, IFN-γ ^7^, IL-1β, and TNF-α and increased IL-10. Improved DAI and histological scores.
[56]	*Bifidobacterium bifidum* 231(BIF 231)	Traditional preparation of fermented food/beverage	Reduced macroscopic and histological damage to the colon and IL-1β, and increased the expression of mRNA and IL-10. It showed similar efficacy to treatment with dexamethasone.
[57]	ID-JPL934 ^8^	Diluted powder or capsule consumption	It showed similar efficacy to sulfasalazine treatment and improved DAI, histological scores, and colon length. Reduced TNF-α, IL-1b, IL-6, and immune cell infiltration.
[44]	*Bifidobacterium longum* (Bif) or VSL # 3	Diluted powder or capsule consumption	Showed similar levels of HMGB19. Improved barrier function, increased ZO-1, occludin, and claudin. VSL#3 reduced colon shortening and dysbiosis.
[24]	*Lactobacillus acidophilus*	Traditional preparation of fermented food/beverage	Prevented weight loss and colon shortening, decreased histological scores, suppressed cytokines IL-6, TNF-α, IL-β, and IL-17, and increased IL-10.
[28]	*Lactobacillus reuteri* F-9-35	Diluted powder or capsule consumption	Reduced weight loss, DAI score, MPO, TNF-α, Cox-211, and IL-6. Improved colon shortening and damage and balanced intestinal microbiota.
[58]	*Lactobacillus acidophilus*, *Clostridium butyricum*	Diluted powder or capsule consumption	Reduced inflammation, IL-4, IL-6, permeability, and neutrophil and macrophage infiltration, and increased ZO-1 expression.
[42]	Bifico ^12^	Diluted powder or capsule consumption	Reduced DAI scores and inflammatory cell infiltration, improved colon length, and increased occludin, claudin, and JAM-1. Pretreatment inhibited TNF α production. Pretreated and treated patients presented more significant intestinal damage.
[60]	*Bifidobacterium longum* 51A	Traditional preparation of fermented food/beverage	Improved permeability, intestinal structure, and colon shortening. Reduced IL-1β and MPO levels.
[61]	*Lactobacillus plantarum* tabaco LB-9	Diluted powder or capsule consumption	It improved colon shortening, reduced DAI score and TNF-α level, and suppressed TNF-α-mediated intestinal epithelial cell (IEC) apoptosis.
[62]	*Lactobacillus plantarum*	Traditional preparation of fermented food/beverage	Reduced weight loss, shortening and damage to the colon, and iNOS ^14^, Cox-2, TNF-α, and IL-6.
[63]	*Lactobacillus fermentum* KBL374 or KBL375	Diluted powder or capsule consumption	Improved weight, DAI score, colon length, histological score, barrier function, and innate immune response. Reduced leukocyte infiltration, IFN-γ, IL-6, TNF-α, and IL-17A and increased IL-10. *L. fermentum* KBL375 improved gut microbiota.
[64]	*Lactobacillus lactis*	Traditional preparation of fermented food/beverage	Mesalazine and LAB ^15^ mixture showed the most effective results by decreasing intestinal lesions and expressing pro-inflammatory cytokines IL-6 and TNF-α.
[65]	*Bacillus licheniformis* Zhengchangsheng^®^	Diluted powder or capsule consumption	Reduced DAI score, colon shortening, weight loss, histological scores, and MPO; increased IL-10; improved the intestinal barrier and modulated the gut microbiota.
[66]	*Lactococcus lactis* ML2018	Diluted powder or capsule consumption	Decreased DAI and histological scores, colon shortening, IL-1β, IL-6, TNF-α, macrophage activity, and colon fibrosis. It protects the intestinal barrier and increases short-chain fatty acids.
[40]	*Lactobacillus plantarum*	Traditional preparation of fermented food/beverage	Increased body weight and colon length, reduced DAI and histological scores, IL-1 β, IL-6, IL-17, and TNF-α, increased IL-10, and modulate gut microbiota.
[67]	*Bifidobacterium infantis*	Traditional preparation of fermented food/beverage	Improved weight, DAI score, and Foxp3, IL-10, and TGF-β1 in the colon. Histological analysis of the colon: best results with 1 × 10^9^ and 1 × 10^8^ CFU doses.
[68]	*Propionibacterium freudenreichii*, *Lactobacillus delbrueckii* and *Streptococcus hermophilus*	Traditional preparation of fermented food/beverage	It decreased colitis severity, histological score, small bowel IgA ^16^, IL-6, IFNγ, and TNFα, increased IL-10, and restored occludin gene expression, but did not prevent colon shortening.
[69]	*Bifidobacterium bifidum* ATCC 29521	Diluted powder or capsule consumption	Reduced DAI, histological scores, MPO, and colon shortening. Improved dysbiosis, increased HMGB1 ^9^ IL-10, ZO-1, MUC-2 ^10^, Claudin-3 and Cadherin-1 mRNAs; decreased TNF-α, IL-1β, and IL-6, COX-2 ^11^.
[47]	*Escherichia coli* Nissle strain 1917 (EcN)	Diluted powder or capsule consumption	Increased the anti-inflammatory effect and IL-10 and ZO-1, decreased DAI score, MPO, TNF-α, and IL-6.
[70]	*Propionibacterium freudenreichii*	Diluted powder or capsule consumption	There were reduced DAI, histological scores, TNF-α, IL-6, and IL-1β, while IL-10 was insignificant. Restored colon length and produced propionate in the feces of rats.
[45]	*Lactobacillus plantarum* CBT LP3	Traditional preparation of fermented food/beverage; diluted powder or capsule consumption	Improved weight loss, colon length, and DAI score. Suppressed TNF-α, IL17, NO, and iNOS. Restored goblet cells and increased IL-10 and TGF-β.
	**Yeasts**		
[59]	*Saccharomyces boulardii*	Diluted powder or capsule consumption	Reduced inflammation, decreased DAI score, weight loss, and histological scores. Increased the expression of claudin-1. Inhibited the expression of HIF-1α and HIF-2α ^13^.
[3]	*Saccharomyces cerevisiae* and *Weissella cibaria*	Traditional preparation of fermented food/beverage; diluted powder or capsule consumption	They reduced weight loss, DAI, histological scores, neutrophil infiltration, TNF-α, IL-6, and IL-8 levels, and lipid peroxidation. They increased colorectal length, antioxidant activities, goblet cells, gene expression for mucins (Mucin-1 and Mucin-2), tight junctions (ZO^−1^, ZO^−2^, Claudin-1, and occludin), and NF-κB: nuclear factor kappa B ^17^, and attenuated dysbiosis.

^1^ DAI: Disease Activity Index; ^2^ MPO: myeloperoxidase; ^3^ GI7: *Lactobacillus acidophilus*, *Lactobacillus plantarum*, *Lactobacillus rhamnosus*, *Lactococcus lactis*, two strains of *Bifidobacterium bifidum*, *Bifidobacterium breve*, and *Streptococcus thermophilus*; ^4^ TNF-α: tumor necrosis factor-alpha; ^5^ TGF-β: transforming growth factor beta; ^6^ LPS: lipopolysaccharide endotoxin; ^7^ IFN-γ: interferon-gamma; ^8^ ID-JPL934: (*Lactobacillus johnsonii* IDCC9203, *Lactobacillus plantarum* IDCC3501 and *Bifidobacterium animalis* subsp. *lactis* IDCC4301; ^9^ HMGB1 (High mobility group Box-1): a protein that stimulates the transcription of pro-inflammatory cytokines; ^10^ MUC: mucins; ^11^ COX-2: cyclooxygenase-2; ^12^ Bifico: (*Bifidobacterium, Lactobacillus*, and *Enterococcus*); ^13^ HIF: hypoxia-inducible factor; ^14^ iNOS: inducible nitric oxide synthase; ^15^ LAB: *Lactobacillus plantarum* CRL 2130, *Streptococcus thermophilus* CRL 808, and *Streptococcus thermophilus* CRL 807; ^16^ Ig: immunoglobulins; ^17^ NF-κB: nuclear factor kappa B.

## Data Availability

No new data were created or analyzed in this study. Data sharing is not applicable to this article.

## Abbreviations

IBD	Inflammatory bowel disease
DAI	Disease Activity Index
UC	Ulcerative colitis
CD	Crohn’s disease
IM	Intestinal microbiota
BVS	Biblioteca Virtual de Saúde
QQL	Quality of life questionnaire
FCAL	Fecal Calprotectin
VN	Varied Number
DSS	Dextran Sodium Sulfate
TNBS	Trinitrobenzenesulfonic Acid

## References

[1] Akkasheh G., Kashani-Poor Z., Tajabadi-Ebrahimi M., Jafari P., Akbari H., Taghizadeh M., Memarzadeh M.R., Asemi Z., Esmailzadeh A. (2016). Clinical and metabolic response to probiotic administration in patients with major depressive disorder: A randomized, double-blind, placebo-controlled trial. Nutrition.

[2] Souza M.M., Belasco A.G.S., Aguilar-Nascimento J.E. (2008). Perfil epidemiológico dos pacientes portadores de doença inflamatória intestinal do estado de Mato Grosso. Revista Brasil. Coloproctol..

[3] Wang Y., Jiang Y., Deng Y., Yi C., Wang Y., Ding M., Liu J., Jin X., Shen L., He Y. (2020). Probiotic Supplements: Hope or Hype?. Front. Microbiol..

[4] Fidélix M., Milenkovic D., Sivieri K., Cesar T. (2020). Microbiota modulation and effects on metabolic biomarkers by orange juice: A controlled clinical trial. Food Funct..

[5] Dore M.P., Rocchi C., Longo N.P., Scanu A.M., Vidili G., Padedda F., Pes G.M. (2019). Effect of Probiotic Use on Adverse Events in Adult Patients with Inflammatory Bowel Disease: A Retrospective Cohort Study. Probiot. Antimicrob. Prot..

[6] da Anunciação T.A., Guedes J.D.S., Tavares P.P.L.G., de Melo Borges F.E., Ferreira D.D., Costa J.A.V., Umsza-Guez M.A., Magalhães-Guedes K.T. (2024). Biological Significance of Probiotic Microorganisms from Kefir and Kombucha: A Review. Microorganisms.

[7] Shor D.B., Bashi T., Lachnish J., Fridkin M., Bizzaro G., Barshak I., Blank M., Shoenfeld Y. (2015). Phosphorylcholine-tuftsin compound prevents development of dextransulfate-sodium-salt-induced murine colitis: Implications for the treatment of human inflammatory bowel disease. J. Autoimmun..

[8] Fan H., Du J., Liu X., Zheng W.W., Zhuang Z.H. (2019). Effects of pentasa-combined probiotics on the microflora structure and prognosis of patients with inflammatory bowel disease. Turk. J. Gastroenterol..

[9] Prisciandaro L., Geier M., Butler R., Cummins A., Howarth G. (2009). Probiotics and their derivatives as treatments for inflammatory bowel disease. Inflammat. Bowel Dis..

[10] Molodecky N.A., Soon I.S., Rabi D.M., Ghali W.A., Ferris M., Chernoff G., Benchimol E.I., Panaccione R., Ghosh S., Barkema H.W. (2012). Increasing incidence and prevalence of the inflammatory bowel diseases with time, based on systematic review. Gastroenterology.

[11] Ng S.C., Bernstein C.N., Vatn M.H., Lakatos P.L., Loftus E.V., Tysk J.C., O’Morain C., Moum B., Colombel J.-F. (2013). Geographical variability and environmental risk factors in inflammatory bowel disease. Gut.

[12] Eom T., Kim Y., Choi C., Sadowsky M., Unno T. (2018). Current understanding of microbiota- and dietary-therapies for treating inflammatory bowel disease. J. Microbiol..

[13] Durchschein F., Petritsch W., Hammer H.F. (2016). Diet therapy for inflammatory bowel diseases: The established and the new. World J. Gastroenterol..

[14] Ordas I., Eckmann L., Talamini M., Baumgart D.C., Sandborn W.J. (2012). Ulcerative colites. Lancet.

[15] Ardizzone S.G., Maconi A., Russo V., Imbesi E., Colombo P., Bianchi G. (2006). Randomised controlled trial of azathioprine and 5-aminosalicylic acid for treatment of steroid-dependent ulcerative colitis. Gut.

[16] da Silva R.N.A., Magalhães-Guedes K.T., de Souza C.O., de Oliveira Alves R.M., Umsza-Guez M.A. (2024). Microbiological and physical-chemical characteristics of pollen and honey from stingless bees: A review. Food Prod. Process. Nutr..

[17] Kostic A.D., Xavier R.J., Gevers D. (2014). The microbiome in inflammatory bowel disease: Current status and the future ahead. Gastroenterology.

[18] Zhao L., Zhang X., Zuo T., Yu J. (2017). The composition of colonic commensal Bacteria according to anatomical localization in colorectal Cancer. Engineering.

[19] He Q., Li X., Liu C., Su L., Xia Z., Li X., Li Y., Li L., Yan T., Feng Q. (2016). Dysbiosis of the fecal microbiota in the TNBS induced Crohn’s disease mouse model. Appl. Microbiol. Biotechnol..

[20] Robles H.V., Madrid A.F.C., Ponce A.G., Olivares A.S., Shibayama M., Betanzos A., Mondragón L.D.V., Nava P., Schnoor M. (2016). Experimental Colitis Is Attenuated by Cardioprotective Diet Supplementation That Reduces Oxidative Stress, Inflammation, and Mucosal Damage. Oxid. Med. Cell. Longev..

[21] Schultz B.M., Paduro C.A., Salazar G.A., Salazarechegarai F.J., Sebastian V.P., Riedel C.A., Kalergis A.M., Alvarez-Lobos M., Bueno S.M. (2017). A potential role of *Salmonella* infection in the onset of inflammatory bowel diseases. Front. Immunol..

[22] Riglar D., Giessen T., Baym M., Kerns S.J., Niederhuber M.J., Bronson R.T., Kotula J.W., Gerber G.K., Way J.C., Silver P.A. (2017). Engineered bacteria can function in the mammalian gut long-term as live diagnostics of inflammation. Nat. Biotechnol..

[23] Neurath M.F. (2014). Cytokines in inflammatory bowel disease. Nat. Rev. Immunol..

[24] Park J.S., Choi J.W., Jhun J., Kwon J.Y., Lee B.I., Yang C.W., Cho M.L. (2018). *Lactobacillus acidophilus* Improves Intestinal Inflammation in an Acute Colitis Mouse Model by Regulation of Th17 and Treg Cell Balance and Fibrosis Development. J. Med. Food.

[25] Mousavi S.N., Saboori S., Asbaghi O. (2020). Effect of daily probiotic yogurt consumption on inflammation: A systematic review and meta-analysis of randomized Controlled Clinical trials. Obes. Med..

[26] Tian P., O’Riordan K.J., Lee Y.-K., Wang G., Zhao J., Zhang H., Cryan J.F., Chen W. (2020). Towards a psychobiotic therapy for depression: *Bifidobacterium brevis* CCFM1025 reverses chonic stress-induce depressive symptoms and gut microbial abnormalities in mice. Neurobiol. Stress.

[27] Cheng L.-H., Liu Y.-W., Wu C.-C., Wang S., Tsai Y.-C. (2019). Psychobiotics in mental health, neurodegenerative and neurodevelopmental disorders. J. Food Drug Anal..

[28] Sun M., Zhang F., Yin X., Cheng B., Zhao C., Wang Y., Ye H. (2018). *Lactobacillus reuteri* F-9-35 Prevents DSS-Induced Colitis by Inhibiting Proinflammatory Gene Expression and Restoring the Gut Microbiota in Mice. J. Food Scien..

[29] Sun H., Park S., Mok J., Seo J., Lee N.D., Yoo B. (2024). Efficacy and Safety of Wilac L Probiotic Complex Isolated from Kimchi on the Regulation of Alcohol and Acetaldehyde Metabolism in Humans. Foods.

[30] Tavares P.P.L.G., Mamona C.T.P., Nascimento R.Q., dos Anjos E.A., de Souza C.O., Almeida R.C.d.C., Mamede M.E.d.O., Magalhães-Guedes K.T. (2023). Non-Conventional Sucrose-Based Substrates: Development of Non-Dairy Kefir Beverages with Probiotic Potential. Fermentation.

[31] Nascimento R.Q., Deamici K.M., Tavares P.P.L.G., de Andrade R.B., Guimarães L.C., Costa J.A.V., Magalhães-Guedes K.T., Druzian J.I., de Souza C.O. (2022). Improving Water Kefir Nutritional Quality via Addition of Viable *Spirulina* Biomass. Bioresour. Technol. Rep..

[32] Magalhães-Guedes K.T., Anunciação T.A., Schwan R.F. (2019). Kombucha and Kefir are foods of the 21st century: An opinion. J. Biotechnol. Bior..

[33] Magalhães-Guedes K.T. (2022). Psychobiotic Therapy: Method to Reinforce the Immune System. Clin. Psychopharmacol. Neurosci..

[34] Pesce M., Seguella L., Del Re A., Lu J., Palenca I., Corpetti C., Rurgo S., Sanseverino W., Sarnelli G., Esposito G. (2022). Next-Generation Probiotics for Inflammatory Bowel Disease. Int. J. Mol. Sci..

[35] Li S., Liu Z., Zhang Q., Su D., Wang P., Li Y., Shi W., Zhang Q. (2024). The Antidiabetic Potential of Probiotics: A Review. Nutrients.

[36] Matsuura N., Kanayama M., Watanabe Y., Yamada H., Lili L., Torii A. (2024). Effect of Personalized Prebiotic and Probiotic Supplements on the Symptoms of Irritable Bowel Syndrome: An Open-Label, Single-Arm, Multicenter Clinical Trial. Nutrients.

[37] Mcgill R., Tukey J.W., Larsen W.A. (1978). Variations of Boxplots. Am. Stat..

[38] Nelson L.S. (1989). Evaluating Overlapping Confidence Intervals. J. Qual. Technol..

[39] Ferreira J.E.V., Pinheiro M.T.S., Santos W.R.S., Maia R.S. (2016). Graphical representation of chemical periodicity of main elements through boxplot. Educ. Química.

[40] Zhang F., Li Y., Wang X., Wang S., Bi D. (2019). The Impact of *Lactobacillus plantarum* on the Gut Microbiota of Mice with DSS-Induced Colitis. BioMed. Res. Inter..

[41] Zhang H.L., Li W.S., Xu D.N., Zheng W.W., Liu Y., Chen J., Liu J. (2016). Mucosa-reparing and microbiota-balancing therapeutic effect of *Bacillus subtilis* alleviates dextrate sulfate sodium-induced ulcerative colitis in mice. Experimen. Ther. Med..

[42] Zhang Y., Zhao X., Zhu Y., Ma J., Ma H., Zhang H. (2018). Probiotic mixture protects dextran sulfate sodium-induced colitis by altering tight junction protein expressions and increasing Tregs. Mediat. Inflammat..

[43] Chen C.L., Hsu P.Y., Pan T.M. (2018). Therapeutic effects of *Lactobacillus paracasei* subsp. *paracasei* NTU 101 powder on dextran sulfate sodium-induced colitis in mice. J. Food Drug Anal..

[44] Chen X., Fu Y., Wang L., Qian W., Zheng F., Hou X. (2018). *Bifidobacterium longum* and VSL# 3^®^ amelioration of TNBS-induced colitis associated with reduced HMGB1 and epithelial barrier impairment. Dev. Comp. Immunol..

[45] Kim D.H., Kim S., Ahn J.B., Kim J.H., Ma H.W., Seo D.H., Cheon J.H. (2020). *Lactobacillus plantarum* CBT LP3 ameliorates colitis via modulating T cells in mice. Inte. J. Medical Microbiol..

[46] Kim M.S., Byun J.S., Yoon Y.S., Yum D.Y., Chung M.J., Lee J.C. (2017). A probiotic combination attenuates experimental colitis through inhibition of innate cytokine production. Benef. Microbes.

[47] Luo X., Song H., Yang J., Han B., Feng Y., Leng Y., Chen Z. (2020). Encapsulation of *Escherichia coli* strain Nissle 1917 in a chitosan―alginate matrix by combining layer-by-layer assembly with CaCl_2_ cross-linking for an effective treatment of inflammatory bowel diseases. Colloids Surf. B. Biointerfaces.

[48] Power S.E., O’toole P.W., Stanton C., Ross R.P., Fitzgerald G.F. (2014). Intestinal microbiota, diet and health. Br. J. Nut..

[49] Xu X.M., Zhang H.J. (2016). miRNAs as new molecular insights into inflammatory bowel disease: Crucial regulators in autoimmunity and inflammation. World J. Gastroenterol..

[50] Baumgart D.C., Sandborn W.J. (2007). Inflammatory bowel disease: Clinical aspects and established and evolving therapies. Lancet.

[51] Nogales A.R., Algieri F., Garrido-Mesa J., Vezza T., Utrilla M.P., Chueca N., Gálvez J. (2018). Intestinal anti-inflammatory effect of the probiotic *Saccharomyces boulardii* in DSS-induced colitis in mice: Impact on microRNAs expression and gut microbiota composition. J. Nutr. Biochem..

[52] Ahl D., Liu H., Schreiber O., Roos S., Phillipson M., Holm L. (2016). *Lactobacillus reuteri* increases mucus thickness and ameliorates dextran sulphate sodium-induced colitis in mice. Acta Physiol..

[53] Thakur B.K., Saha P., Banik G., Saha D.R., Grover S., Batish V.K., Das S. (2016). Live and heat-killed probiotic *Lactobacillus casei* Lbs2 protects from experimental colitis through Toll-like receptor 2-dependent induction of T-regulatory response. Inter. Immunopharmacol..

[54] Celiberto L.S., Bedani R., Dejani N.N., Medeiros I.A., Sampaio Z.J.A., Sampaio Z.J.A., Spolidorio L.C., Cavallini D.C.U. (2017). Effect of a probiotic beverage consumption (*Enterococcus faecium* CRL 183 and *Bifidobacterium longum* ATCC 15707) in rats with chemically induced colitis. PLoS ONE.

[55] Park J.S., Joe I., Rhee P.D., Jeong C.-S., Jeong G. (2017). A lactic acid bacterium isolated from kimchi ameliorates intestinal inflammation in DSS-induced colitis. J. Microbiol..

[56] Satish K.C.S.V., Kondal R.K., Boobalan G., Gopala R.A., Sudha R.C.C., Vinoth A., Srinivasa R.G. (2017). Immunomodulatory effects of *Bifidobacterium bifidum* 231 on trinitrobenzenesulfonic acid-induced ulcerative colitis in rats. Res. Vet. Sci..

[57] Je I.G., Lee D.G., Jeong D.G., Hong D., Yoon J.M., Moon J.S., Park S. (2018). The Probiotic, ID-JPL934, Attenuates Dextran Sulfate Sodium-Induced Colitis in Mice Through Inhibition of Proinflammatory Cytokines Expression. J. Med. Food.

[58] Wang Y., Gu Y., Fang K., Mao K., Dou J., Fan H., Wang H. (2018). *Lactobacillus acidophilus* and *Clostridium butyricum* ameliorate colitis in murine by strengthening the gut barrier function and decreasing inflammatory factors. Benef. Microbes.

[59] Zhou H., Zhang H., Guan L., Zhang Y., Li Y., Sun M. (2018). Mechanism and therapeutic effects of *Saccharomyces boulardii* on experimental colitis in mice. Mol. Med. Rep..

[60] Abrantes F.A., Nascimento B.B., Andrade M.E.R., Barros P.A.V., Cartelle C.T., Martins F.S., Cardoso V.N. (2019). Treatment with *Bifidobacterium longum* 51A attenuates intestinal damage and inflammatory response in experimental colitis. Benef. Microbes.

[61] Chae J.M., Chang M.H., Heo W., Cho H.T., Lee D.H., Hwang B.B., Kim Y.J. (2019). LB-9, Novel Probiotic Lactic Acid Bacteria, Ameliorates Dextran Sodium Sulfate-Induced Colitis in Mice by Inhibiting TNF-α-Mediated Apoptosis of Intestinal Epithelial. J. Med. Food.

[62] Choi S.H., Lee S.H., Kim M.G., Lee H.J., Kim G.B. (2019). *Lactobacillus plantarum* CAU1055 ameliorates inflammation in lipopolysaccharide-induced RAW264. 7 cells and a dextran sulfate sodium–induced colitis. J. Dairy Sci..

[63] Jang Y.J., Kim W.K., Han D.H., Lee K., Ko G. (2019). *Lactobacillus fermentum* species ameliorate dextran sulfate sodium-induced colitis by regulating the immune response and altering gut microbiota. Gut Microbes.

[64] Levit R., Savoy G.G., LeBlanc M.A., LeBlanc J.G. (2019). Beneficial effect of a mixture of vitamin-producing and immune-modulating lactic acid bacteria as adjuvant for therapy in a recurrent mouse colitis model. Appl. Microbiol. Biotechnol..

[65] Li Y., Liu M., Zhou J., Hou B., Su X., Liu Z., Li M. (2019). *Bacillus licheniformis* Zhengchangsheng^®^ attenuates DSS-induced colitis and modulates the gut microbiota in mice. Benef. Microbes.

[66] Liu M.L., Zhang X., Hao Y., Ding J., Shen J., Xue Z., Wang N. (2019). Protective effects of a novel probiotic strain, *Lactococcus lactis* ML2018, in colitis: In vivo and in vitro evidence. Food Funct..

[67] Zhou L., Liu D., Xie Y., Yao X., Li Y. (2019). *Bifidobacterium infantis* Induces Protective Colonic PD-L1 and Foxp3 Regulatory T Cells in an Acute Murine Experimental Model of Inflammatory Bowel Disease. J. Chest. Surg..

[68] Rabah H., Carmo F.L.R., Carvalho R.D.O., Cordeiro B.F., Silva S.H., Oliveira E.R., Jan G. (2020). Beneficial propionibacteria within a probiotic emmental cheese: Impact on dextran sodium sulphate-induced colitis in mice. Microorganisms.

[69] Din A.U., Hassan A., Zhu Y., Zhang K., Wang Y., Li T., Wang G. (2020). Inhibitory effect of *Bifidobacterium bifidum* ATCC 29521 on colitis and its mechanism. J. Nutrit. Biochem..

[70] Ma S., Yeom J., Lim Y.H. (2020). Dairy *Propionibacterium freudenreichii* ameliorates acute colitis by stimulating MUC2 expression in intestinal goblet cell in a DSS-induced colitis rat model. Sci. Rep..

[71] Bjarnason I., Sission G., Hayee B. (2019). A randomised, double-blind, placebo-controlled trial of a multi-strain probiotic in patients with asymptomatic ulcerative colitis and Crohn’s disease. Inflammopharmacology.

[72] Dieleman L.A., Palmen M.J., Akol H., Bloemena E., Peña A.S., Meuwissen S.G., Rees E.P.V. (1998). Chronic experimental colitis induced by dextran sulphate sodium (DSS) is characterized by Th1 and Th2 cytokines. Clin. Exp. Immunol..

[73] Indira M., Venkateswarulu T.C., Abraham P.K., Nazneen B.M., Krupanidhi S. (2019). Bioactive molecules of probiotic bacteria and their mechanism of action: A review. 3 Biotech.

[74] Barra M., Danino T., Garrido D. (2020). Engineered Probiotics for Detection and Treatment of Inflammatory Intestinal Diseases. Front. Bioeng. Biotechnol..

[75] Daeffler K.N., Galley J.D., Sheth R.U., Ortiz-Velez L.C., Bibb C.O., Shroyer N.F., Britton R.A., Tabor J.J. (2017). Engineering bacterial thiosulfate and tetra-thionate sensors for detecting gut inflammation. Mol. Syst. Biol..

[76] Nejad H.R., Oliveira B.C.M., Sadeqi A., Dehkharghani A., Kondova I., Langermans J.A.M., Guasto J.S., Tzipori S., Widmer G., Sonkusale S.R. (2019). Ingestible osmotic pill for in vivo sampling of gut microbiomes. Adv. Intell. Syst.-Ger..

[77] Goh S., Roberts A.P., Mullany P. (2016). Phage transduction. Clostridium difficile: Methods and Protocols.

[78] Virolle C., Goldlust K., Djermoun S., Bigot S., Lesterlin C. (2020). Plasmid transfer by conjugation in Gram-negative bacteria: From the cellular to the community level. Genes.

[79] Qi H., Yu L., Li Y.Z., Cai M., He J.Z., Liu J.Y., Hao L., Xu H., Qiao M. (2022). Developing multi-copy chromosomal integration strategies for heterologous biosynthesis of caffeic acid in *Saccharomyces cerevisiae*. Front. Microbiol..

[80] Ma J., Lyu Y., Liu X., Jia X., Cui F., Wu X., Deng S., Yue C. (2022). Engineered probiotics. Microb. Cell Fact..

[81] Magalhães K.T., Pereira M.A., Dragone G., Nicolau A., Domingues L., Teixeira J.A., Silva J.B.A., Schwan R.F. (2010). Production of fermented cheese whey-based beverage using kefir grains as starter culture: Evaluation of morphological and microbial variations. Bioresour. Technol..

[82] Yan X., Liu X.Y., Zhang D., Zhang Y.D., Li Z.H., Liu X., Wu F., Chen G.-Q. (2021). Construction of a sustainable 3-hydroxybutyrate-producing probiotic *Escherichia coli* for treatment of colitis. Cell. Mol. Immunol..

[83] De la Rosa González A., Guerra-Ojeda S., Camacho-Villa M.A., Valls A., Alegre E., Quintero-Bernal R., Martorell P., Chenoll E., Serna-García M., Mauricio M.D. (2024). Effect of Probiotics on Gastrointestinal Health Through the Aryl Hydrocarbon Receptor Pathway: A Systematic Review. Foods.

[84] Bentahar M.C., Benabdelmoumene D., Robert V., Dahmouni S., Qadi W.S.M., Bengharbi Z., Langella P., Benbouziane B., Al-Olayan E., Dawoud E.A.D. (2024). Evaluation of Probiotic Potential and Functional Properties of *Lactobacillus* Strains Isolated from Dhan, Traditional Algerian Goat Milk Butter. Foods.

[85] Zhang A., Ou M., Wu P., Zheng K., Zhang H., Yu Y., Guo Y., Zhang T., Pan D., Wu Z. (2024). Coupled Effect of Nutritional Food Molecules and *Lactobacillus reuteri* Surface Protein Interaction on the Bacterial Gastrointestinal Tolerance. Foods.

[86] Shen X., Ma C., Yang Y., Liu X., Wang B., Wang Y., Zhang G., Bian X., Zhang N. (2024). The Role and Mechanism of Probiotics Supplementation in Blood Glucose Regulation: A Review. Foods.

[87] Song J. (2024). Functional Properties of Probiotics in Food Sources. Foods.

[88] Magalhães-Guedes K.T. (2020). The Dialogue between the Intestine-brain Axis: What is the Role of Probiotics?. Asian Food Sci. J..

[89] López-Almada G., Mejía-León M.E., Salazar-López N.J. (2024). Probiotic, Postbiotic, and Paraprobiotic Effects of *Lactobacillus rhamnosus* as a Modulator of Obesity-Associated Factors. Foods.

